# Intermittent theta-burst stimulation combined with physical therapy as an optimal rehabilitation in Parkinson’s disease: study protocol for a randomised, double-blind, controlled trial

**DOI:** 10.1186/s13063-023-07425-7

**Published:** 2023-06-16

**Authors:** Zhao-hui Jin, Yi-xuan Wang, De-tao Meng, Yi Qin, Yi-nan Duan, Jin-ping Fang, Rui-dan Wang, Yan-jun Liu, Cui Liu, Ping Wang, Hong-jiao Yan, Yi Zhen, Xia An, Ke-ke Chen, Xin Yu, Diyang Lyu, Xiao-Yan Yan, Bo-yan Fang

**Affiliations:** 1 Parkinson Medical Center, Beijing Rehabilitation Hospital, Capital Medical University, Badachu, Xixiazhuang, Shijingshan District, Bejing, 100144 China; 2 Capital Medical University, Beijing, China; 3 Peking University Clinical Research Institute, Peking University First Hospital, Beijing, China

**Keywords:** Intermittent theta-burst stimulation, Parkinson’s disease, Physiotherapy, Randomised controlled trial

## Abstract

**Background:**

First-line rehabilitative strategies to improve motor deficits are based on functional training (physical or occupational therapy), which has been demonstrated to facilitate neural reorganisation. Accumulating evidence suggests that non-invasive brain stimulation techniques, such as repetitive TMS (rTMS), may enhance neuroplasticity, thereby facilitating neural reorganisation and recovery from Parkinson’s disease. Evidence also shows that intermittent theta-burst stimulation (iTBS) can improve motor function and quality of life in patients by promoting the excitability and neural remodelling of cerebral cortex. We aimed to combine iTBS stimulation with physiotherapy to improve the rehabilitation effect compared to physiotherapy alone in patients with Parkinson’s disease.

**Methods:**

This randomised, double-blind clinical trial will enrol 50 Parkinson’s disease patients aged 45–70 years with Hoehn and Yahr scale scores of 1–3. Patients are randomly assigned to either the iTBS + physiotherapy or sham-iTBS + physiotherapy group. The trial consists of a 2-week double-blind treatment period and a 24-week follow-up period. iTBS and sham-iTBS will be administered twice daily for 10 days based on physiotherapy. The primary outcome will be the third part of Movement Disorders–Unified Parkinson’s Disease Rating Scale (MDS-UPDRS III) from the baseline to the first 2 days following completion hospitalised intervention. The secondary outcome will be 39-item Parkinson’s Disease Questionnaire (PDQ-39) at 4 weeks, 12 weeks and 24 weeks after intervention. Tertiary outcomes are clinical evaluations and mechanism study outcomes such as NMSS, 6MWD, 10MT, TUG, BBS, MRI, and EEG, the length of time between the drug needs to be adjusted when symptoms fluctuate.

**Discussion:**

The aim of this study is to demonstrate that iTBS can promote overall function and quality of life in Parkinson’s disease patients using physiotherapy and that this efficacy may be associated with altered neuroplasticity in exercise-related brain regions. The iTBS combined with physiotherapy training model will be evaluated during a 6-month follow-up period. With significant improvement in quality of life and motor function, iTBS combined with physiotherapy can be considered as a first-line rehabilitation option for Parkinson’s disease. The potential of iTBS to enhance neuroplasticity in the brain should have a more positive impact in increasing the generality and efficiency of physiotherapy, improving the quality of life and overall functional status of patients with Parkinson’s disease.

**Trial registration:**

Chinese Clinical Trial Registry ChiCTR2200056581. Registered on 8 February 2022.

**Supplementary Information:**

The online version contains supplementary material available at 10.1186/s13063-023-07425-7.

## Background

Parkinson’s disease (PD) is the second most prevalent age-related, progressive, neurodegenerative disorder, with both motor and non-motor symptoms that substantially impact the quality of life and economic burden of older adults [1, 2]. The clinical features of PD include motor [3, 4] and non-motor symptoms. Gait and posture disorders remain the most discriminating features of the pathology [5, 6] and all the disorders can disable patients and severely limit their quality of life [6-8]. Although non-motor symptoms can reduce a patient’s quality of life, treatment of PD primarily focuses on improving motor symptoms [9].

One review showed that many studies have been undertaken to investigate the effectiveness of repetitive transcranial magnetic stimulation (rTMS) for PD patients with motor dysfunction [10]. rTMS therapy has been reported to be an effective treatment for motor symptoms of PD, and multi-session high frequency (HF) stimulation on M1 can be an optimal stimulation protocol. rTMS can directly affect cortical excitability at the site of stimulation and may also affect the excitability of other brain regions associated with the site of stimulation through the cortico-striatum-thalamus-cortical circuitry. It has been suggested that rTMS might influence cortical excitability and, thus, brain function and can be applied in bursts of HF (≥ 5 Hz) or continuous trains of low frequency (LF) (≤ 1 Hz). In general, the current generated by the magnet through the time-varying magnetic field acts on the cerebral cortex, producing neuroplasticity changes similar to long-term enhancement (LTP) or depression. HF-rTMS can enhance excitability in the cortex, and LF-rTMS can reduce excitability [11-13].

Theta-burst stimulation (TBS) is another form of rTMS protocol with HF and low‐intensity stimulation, which may facilitate induction of plasticity mechanisms [14], and uses a short stimulation duration, low stimulation pulse intensity, and a possibility to improve rTMS efficiency [15]. When TBS is delivered continuously (cTBS), it decreases cortical excitability; whereas intermittent theta-burst stimulation (iTBS) increases cortical excitability [16]. Because TBS may have fewer adverse effects, such as seizures, impairment of hearing and cognition function [17], and shorter time of single intervention (within several minutes) compared with traditional rTMS, in recent years, an increasing number of studies have begun to explore the therapeutic effect of TBS on motor and non-motor symptoms in patients with PD (PWP) [18-27].

Physiotherapy has been advocated for PD patients. There is substantial evidence that physical therapy can be beneficial in improving walking, muscle strength and balance or reducing falls [28-30]. Thus, physiotherapy (PT) aims to protect, enhance or restore movement and bodily functions that are impaired or threatened by disease, injury and disability. Training techniques include active exercise modalities such as aerobic endurance and muscle strength training, cueing techniques and cognitive motor strategies to improve limitations in fitness, gait, balance, posture and transfer [31]. The focus of these studies has been on motor difficulties such as gait impairment and balance problems. There are numerous studies showing that rTMS combined with rehabilitation therapy has a good effect on stroke, schizophrenia and other diseases, especially in the improvement of upper limb motor function [32], dysphagia [33], aphasia function [34] and cognitive function [35] after stroke.

This trial aims to estimate the efficacy and effectiveness of iTBS combined with physiotherapy. We aim to explore the application of iTBS and new PT rehabilitation programmes in Parkinson’s disease, which may provide evidence for new rehabilitation models with higher economic benefit ratio of PD.

## Methods

### Study design

The study will be a double-blind, randomised, placebo-controlled trial. This protocol is reported in accordance with the Standard Protocol Items: Recommendations for Interventional Trials guidelines [36]. We hypothesise that iTBS combined with physiotherapy is more efficient and effective than physiotherapy alone for the treatment of PD.

**Fig. 1:**
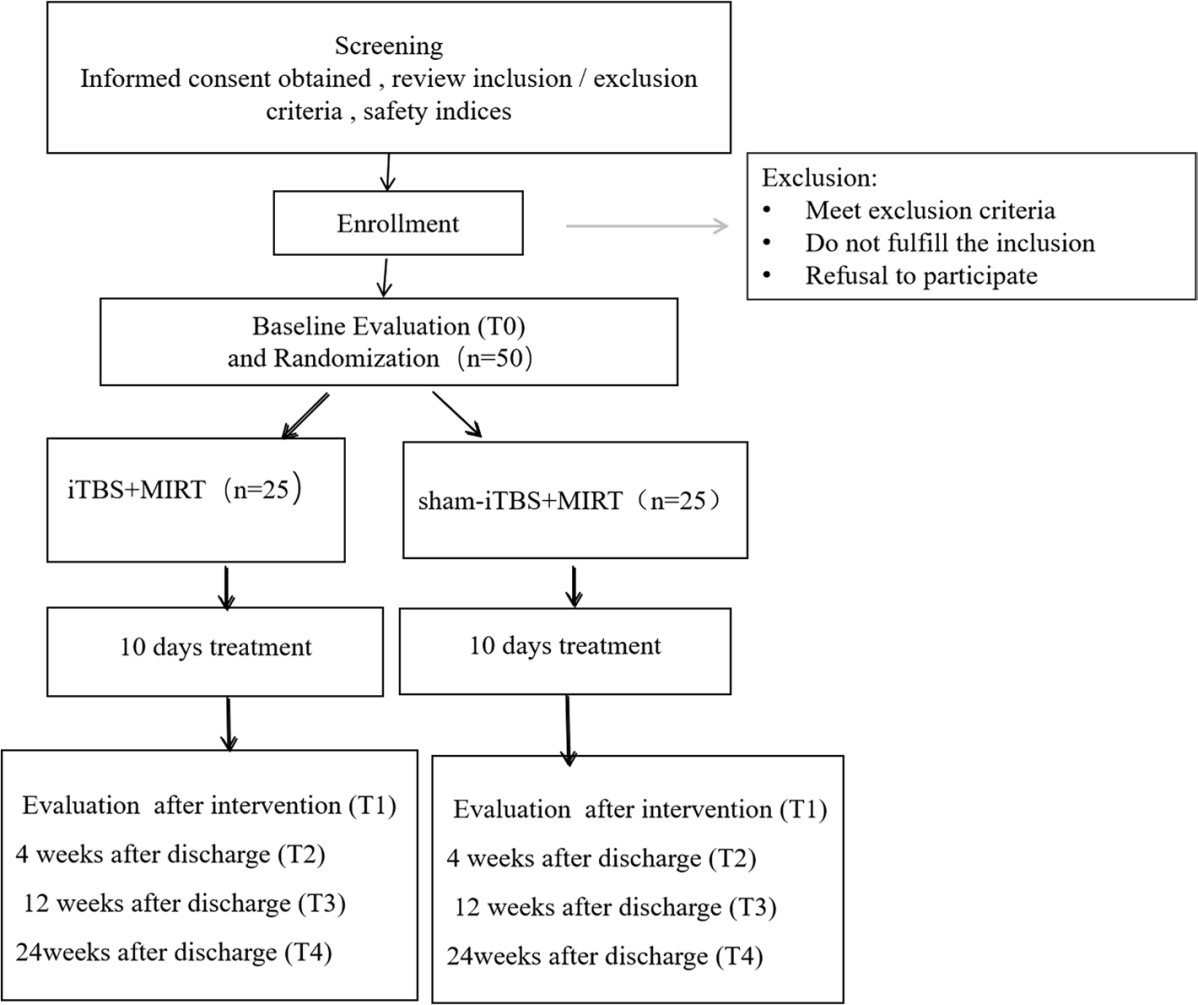
Study design flow chart. The 26-week study will include baseline evaluation (T0) for 2 days, an intervention time of 10 days, and efficacy will be assessed after intervention for 2 days (T1), 4 weeks (T2), 12 weeks (T3) and 24 weeks (T4)

The protocol adheres to the guidelines outlined by the Helsinki Declaration and Measures for Ethical Review of Biomedical Research Involving Humans and has been approved by the Ethics Committee of Beijing Hospital, Capital Medical University. The trial will be conducted under the supervision of the Department of Scientific Research Management of the Beijing Rehabilitation Hospital, Capital Medical University. All participants provided written informed consent before participation. This study was registered in the Chinese Clinical Trial Registry (ChiCTR2200056581). The total study duration for one patient will be 26 weeks, with baseline evaluation (T0) for 2 days and an intervention time of 10 days, and efficacy will be assessed after intervention for 2 days (T1), 4 weeks (T2), 12 weeks (T3) and 24 weeks (T4) (Fig. 1).

### Participants

PD patients will be recruited through the outpatient clinic of Beijing Rehabilitation Hospital, Capital Medical University, as well as through the hospital website. Recruitment will continue in 2023, and participants will be randomly assigned (at a 1:1 ratio) to the iTBS + PT or sham-iTBS + PT group, with 25 participants in each group. Patients with appointments will first be screened by telephone to determine their qualification, and then to go to the outpatient clinic to conduct on-site screening. The recruitment and intervention of the trial group will be carried out in the Beijing Rehabilitation Hospital, Capital Medical University. The follow-up evaluation at each time point will be carried out online by the electronic data capture (EDC) system.

All participants must sign a written informed consent form. Participation is voluntary and patients can leave the study at any time. All of the data will be anonymised. We will establish multiple communication channels to maintain close contact with participants.

### Inclusion criteria

- Conformity with the diagnostic criteria of idiopathic PD according to the 2015 Movement Disorder Society (MDS) criteria [37]
- Age of 45–70 years and primary school education level and above
- Patients that are classified as 1–3 according to the Hoehn and Yahr scale [38, 39]
- Patients prescribed a type and dosage of drugs used for more than 2 weeks that has not been adjusted in the short term
- Those who can walk without assistance (including assistive devices) and need no help from others in daily life
- Subjects that consciously abide by the ward management regulations and epidemic prevention requirements and pass the admission and epidemic prevention screening as required
- Those who voluntarily sign the informed consent form and confirm that they will be able to complete the treatment

### Exclusion criteria

- Patients who do not take their medication under the guidance of a doctor or cannot take their medication regularly
- Subjects with visual or hearing impairment, cardiovascular or cerebrovascular diseases, and respiratory system diseases
- Those with secondary Parkinson syndrome or Parkinsonism-plus syndrome caused by drugs, encephalitis, metabolic diseases, stroke, carbon monoxide poisoning, brain tumours or other neurologic degenerative diseases
- Patients who are unable to cooperate throughout the study because of drug abuse or poor adherence according to the investigators’ judgement
- Subjects who have participated in other studies in the 2 weeks prior or are currently participating in other clinical trials
- Pregnant or lactating women
- Insufficient compliance
- Patients with cognitive, motor, speech and other impairments caused by other nervous system diseases
- A history of epilepsy or other contraindications to transcranial magnetic stimulation
- Subjects who are unwilling or unable to participate in MRI examinations (such as those with claustrophobia or non-MRI-compatible implants in the body)
- Severe cognitive impairment or audio-visual impairment
- Serious concomitant diseases such as heart, lung, liver and kidney insufficiency
- Those who are participating in other clinical trials or are going to undergo DBS surgery or participate in other clinical trials in the next 12 months
- Patients who are not willing to sign the informed consent form
- Those who do not meet or do not cooperate with the requirements of epidemic prevention

### Dropping criteria

- Patients who do not finish the training among the two groups
- Aggravation of the disease that will require a replacement of drugs

### Randomisation

A researcher who is not involved in the assessments or stimulation will perform the randomisation of the patients. We will use the complete randomisation function of SPSS 26.0 statistical software (IBM, Chicago, IL, USA) to obtain a table of random numbers, and SPSS Visual Binning function has programmed its system to pull from spreadsheets of random numbers to determine assignment. There is no human involvement, and the process is fully concealed from both study investigators and prospective participants until the study arm is assigned.

### Blinding

Patients and clinical researchers will be kept unaware of the course of treatment until the study is completed. The doctors who implement the iTBS and the researchers in charge of randomising the group will know the packet information but will not communicate with the patients. To ensure double blinding, we will instruct patients to not discuss their treatment groups with other patients or staff. When serious adverse events are encountered that require immediate unblinding, the person in charge of the scientific research management department, the project leader of the data management department and the statistician will do so jointly and record the unblinding process in detail. Unblinding will only reveal the kind of patient treatment with a random number and does not affect the blinding of other participants.

### Follow-up

Treatment outcomes will be estimated at baseline (week 0) and after intervention for 2 days (T1), 4 weeks (T2), 12 weeks (T3) and 24 weeks (T4). We will call each participant to keep in contact and remind them of the next interview to avoid being lost to follow-up.

### Intervention

After baseline estimations (days 1–2), patients will receive active or sham-iTBS for 10 consecutive days (days 3–12).

### iTBS + PT group (study group)

**Fig. 2:**
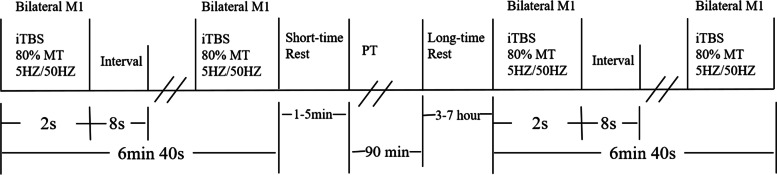
The iTBS + PT group

During each treatment, the patient will receive 3 min and 20 s of iTBS stimulation, twice daily, and PT once a day (Fig. 2).

ITBS (Magstim Rapid^2^, Whitland, UK) will use standard, internationally approved parameters: 80% resting movement threshold (RMT) stimulation intensity; triplet 50 Hz bursts, repeated at 5 Hz for 2 s on and 8 s off, 10 times; 600 pulses per session; for a total duration of 3 min 20 s.

For the RMT measurement, the centre of the figure-of-eight coil is aimed at the base of the scalp of the cerebral cortex movement area, the patient is told to completely relax, and the transcranial magnetic stimulation output intensity is gradually adjusted. Ten consecutive single stimulations can induce five movement evoked potentials with an amplitude greater than 50 V minimum stimulation intensity; or 10 stimulations, at least five times, will cause the minimum intensity of the left thumb muscle contraction [40]. The frameless stereo optical tracking and navigation system is used for positioning. We will conduct dynamic real-time monitoring throughout the treatment process to ensure the accuracy of the target. To obtain stimulation target M1, all participants will undergo MRI brain scans prior to the study: the target is a spherical image centred on the centre point of the M1 surface in a standard brain template based on T1-weighted anatomical magnetic resonance structural images. The target of M1 is transformed into an image of the brain structure of each individual, which is then introduced into a frameless neuronavigation system (Brainsight, Brainbox Ltd, Cardiff, UK).

The coil remains horizontal and tangential to the skull, pointing forward, with its centre point overlapping the target of M1 [41, 42]. Initial treatment consists of twice-daily sessions (on week days; i.e., five sessions a week).

PT is performed in the hospital, consisting of four types of treatments of 30 min each day for 10 days, 6 days of PT consist of session 1, session 2 and session 3 at weekdays; the other 4 days of PT consist of session 4 at weekends. The first treatment is one-on-one physical therapist treatment. It includes cardiovascular warm-up activities, active and passive movements to improve the range of motion of joints, stretching of the abdominal muscles, strengthening of the paraspinal muscles, changes in posture, and movements of balance and posture control. The second session will use a Biofeedback Balance Instrument to improve balance, 30 min a day. Balance training has been successfully used to improve balance in neuropathic patients [43-46], which initiates stabilising responses to postural perturbations and is likely to improve trunk control [47]. It is certainly related to balance and equilibrium and is appropriate to challenge the sensorimotor integration mechanisms [48].

The third session will use a variety of special equipment to improve gait and balance: a stable treadmill with biofeedback (multiple instruction persistence designed according to the principle of virtual reality); walking and gait training under the interference of virtual multiple obstacles and exogenous signals (C-mill); and a treadmill enhancement (treadmill training with auditory cues and feedback). The maximum treadmill speed is 3.5 km/h; patients train on a treadmill for no more than 30 min a day. The fourth session will use gymnastics designed by the physiotherapists Hongjiao Yan specifically for patients with Parkinson's disease. This gymnastics can improve the patient’s balance, gait and mobility**.**

### Sham-iTBS + PT group (control group)

**Fig. 3:**
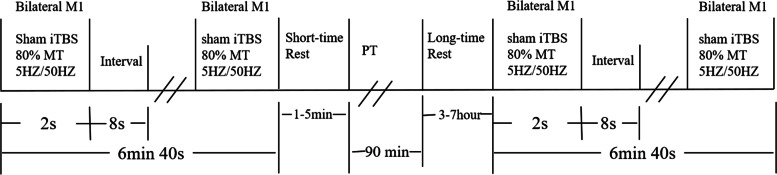
The sham-iTBS + PT group

During each treatment, the patient performs approximately 3 min and 20 s of sham-iTBS, twice daily, and PT once a day (Fig. 3). On sham stimulation, an eight-word false stimulation coil that can simulate sound and touch, such as Air film, which does not induce any significant physiological transcranial effect on the brain, will be used. The sham parameters will be set to 80% RMT, 50 Hz, 3 intra pulses, 5 Hz and 200 stimulus clusters [49]_._ Treatment will be twice daily. The PT training method and duration are as for the study group.

If the patient suffers an unbearable headache, we will reduce the intensity of the intervention to 40 Hz or below; if more serious side effects occur, we will choose the parameters suitable for the patient’s tolerance or stop the treatment altogether. Doctors and therapists will report adverse events during the intervention in writing to the study group in the EDC system. Falls, injuries and harmful adverse events will also be reported due to the rules and regulations of Beijing Rehabilitation Hospital, Capital Medical University.

### Primary outcome measures

The primary outcome will be the change in the third part of Movement Disorders–Unified Parkinson’s Disease Rating Scale (MDS-UPDRS III) from the baseline to the first 2 days following completion hospitalised intervention. MDS-UPDRS III [50] contains 18 items and each item was rated 0 to 4 points. The higher the scores, the poorer the motor symptoms (MS).

### Secondary outcome

The secondary outcomes measures were minimal clinically meaningful response (− 4.72) on 39-item Parkinson’s disease questionnaire (PDQ-39) at 4 weeks, 12 weeks, and 24 weeks after intervention [51]. The PDQ-39 is designed to assess the quality of life of people with PD and consists of 39 programmes which are divided into eight core issues: communication, social support, disease stigma, mobility, emotional health, ability to perform daily living, cognition and physical discomfort. Patients are required to indicate the frequency of each corresponding event in the previous month. The final results of the scale include one global index score, eight sub core scores and a weighted percentage of problem severity. Comparative studies of other quality of life (QoL) scales have shown that PDQ-39 has the highest sensitivity to QoL evaluation in patients with PD, as it presents a higher Cronbach’s alpha index (0.76–0.93) regarding the correlation of the emotional changes of the people with PD with their status of health [52]. This concludes with the necessity of similar highly confident reliability use of the appropriate evaluation scales between clinical trials.

### Tertiary outcomes are clinical evaluations and mechanism study outcomes

Patients in both groups will be evaluated with the following clinical, functional and motor scales to reflect the clinical progress, motor performance, balance ability, fall risk and other functional disorders of PD patients:

- The NMSS [53] allows quantitative evaluation of NMS of PD
- Walking ability: 6-min walk distance (6MWD) measures the distance that a patient can quickly walk on a flat, hard surface in a period of 6 min [54]. The 10 Metre Walk (10MT) is used to assess walking speed, including comfortable and fast gait speeds [55]
- Balance and posture control: timed up and go (TUG) is a measure of functional mobility, which indirectly reflects dynamic balance [56]. Berg balance scale (BBS) is used to evaluate the balance control [57]. Five times sit to stand (FTSTS) relates to balance and bradykinesia in PD [58]
- The length of time between the drug needs to be adjusted when symptoms fluctuate.

#### Mechanism research

Changes in brain plasticity in different periods before and after rehabilitation intervention will be determined by functional electroencephalogram (EEG) and magnetic resonance imaging (fMRI).

##### Electrophysiological recordings

The NeuroScan ERP recording system (Neuro Scan, Sterling, VA, USA) will be used to record the EEG data of 64 scalp elastic caps, which are expanded according to the international 10–20 system [59]. During the EEG recording, each electrode is referenced to the left mastoid. When recording online, the scalp resistance of each electrode is kept below 10 kΩ. The filtered wideband for recording is 0.05–100 Hz. EEG will be continuously sampled at a frequency of 500 Hz.

##### fMRI protocol

MR Data Acquisition Brain imaging is performed using an optimised protocol, using the 3.0-T MRI scanner (GE pioneer) [60]. The acquisition parameters for functional data are as follows: TR = 2000 ms; TE = 21 ms; FA = 90°; FOV = 240 × 240 mm; matrix = 64 × 64; slice thickness = 4.0 mm; and voxel size = 3.75 × 3.75 × 4.0 mm. During the Rs-fMRI scan, participants are instructed to relax with their eyes closed, but to not fall asleep. T1-weighted structural images are acquired using a three-dimensional magnetisation prepared rapid acquisition gradient-echo sequence (TR = 2530 ms, TE = 2.93 ms, FA = 7°, FOV = 256 mm, a 256 × 256 matrix, and a slice thickness of 1.0 mm) [61].

### Data collection

**Fig. 4:**
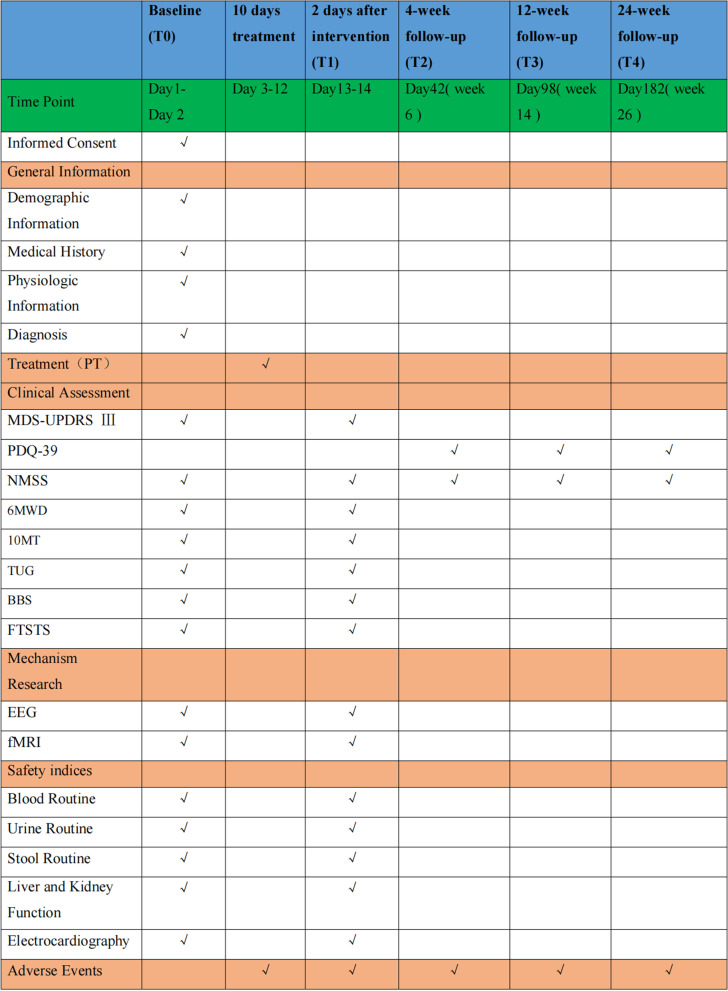
Time schedule of enrolment, interventions and assessments. At baseline (T0), subjects will receive and sign informed consent, and general patient information will be collected. The MDS-UPDRS III will be used to comprehensively evaluate MS of PWP at T0 and T1. The PDQ-39 evaluates the quality of life of patients with PD (PWP) at 4 weeks, 12 weeks and 24 weeks after intervention. The NMSS allows quantitative evaluation of NMS of PD. The changes in brain plasticity in different periods before and after rehabilitation intervention (at T0 and T1) were determined by magnetic resonance imaging (fMRI) and electroencephalogram (EEG). The 6-min walk distance (6MWD), 10-m walk (10MT), TUG, BBS, FTSTS for balance and posture control, and walking ability will be evaluated at T0 and T1. Any AEs will be recorded during the research. Safety indices will also be tested at T0 and T1. AEs will be recorded during the research

At baseline, the following data will be collected: gender, age, occupation, education level, current medication status, detailed physical and neurological examinations. Inclusion and exclusion criteria will be evaluated. MDS-UPDRS III will be evaluated at T0 and T1. The PDQ-39 will be evaluated at 4 weeks, 12 weeks and 24 weeks after intervention. NMSS will be assessed at T0, T1, T2, T3 and T4. The 6MWD, 10MT, TUG, BBS, MRI and EEG will be scanned at T0 and T1. All the above assessments are evaluated blindly by the raters, who are unaware of the treatment methods used by the patients (Figs. 2 and 4).

Safety indices such as blood routine, urine routine, stool routine, liver and kidney function, and electrocardiogram will also be tested at T0 and T1. Adverse events will be recorded during the research. On the consent form, participants will be asked if they agree to use of their data should they choose to withdraw from the trial. Participants will also be asked for permission for the research team to share relevant data with people from the Universities taking part in the research or from regulatory authorities, where relevant. With patient consent, biological samples such as blood, urine, faeces and saliva will be collected at T0 and T1 for molecular biological analysis. Before the start of the study, investigators will receive standardised training on the data collection strategy and guidelines for the different scales used to assess outcomes. During the trial, data will be collected by trained researchers to prevent bias. If the subjects experience the “on–off” phenomenon, the data collection should be conducted during the “on” period. All treatments and evaluations will be conducted at the same period after levodopa taken before and after the intervention to limit the effects of drug fluctuations.

### Data management

An EDC system that relies on the clinical research data platform of the Parkinson Medical Center, Beijing Rehabilitation Hospital will be used for data entry and management [61]. The data entry will be carried out by data collectors. Participant information will be stored in the EDC system, which investigators can access using a password. The completeness of case report forms and consistency between the code and the subject’s screening entry list will be verified. Data will be validated after entry into the EDC system, and the original case will be reviewed based on these results. Final data will be exported as an Excel (Microsoft, Redmond, WA, USA) spreadsheet. Fang Boyan and Yan Xiaoyan will conduct an interim analysis and make a final decision on whether to suspend the trial based on the interim results. Boyan Fang will have access to the final trial dataset, and only authorised researchers can access the data.

### Data monitoring

We have already established an independent data and safety monitoring board (DSMB) for this study that is independent of the sponsor, consisting of a rehabilitation physician specialist, a neurologist and a statistician. The DSMB will oversee whether the trial follows the study design and standard guidelines. The neurologist will assess, manage and classify all adverse events (AEs). All serious AEs (SAEs) will need to be reported to the main researchers, the ethics committees and the DSMB, within 24 h of occurrence. The DSMB and the main researchers will discuss such issues. The ethics committees and the DSMB will have the right to terminate the trial if they deem that such action is appropriate. The role of the statistician is to review the safety data. The DSMB meet to review trial conduct every 3 months. The DSMB meet to review conduct throughout the trial period. The DSMB is also responsible for monitoring the timeliness, completeness and accuracy of data, and urging researchers to improve data reporting and correction in a timely manner. DSMB members are independent of the sponsor and have no competing interests. There is also a coordinating centre that running the trial day-to-day and providing organisational support every day.

Any amendment to the protocol will require approval by the ethics committee before implementation. Participant personal information will be kept private; only relevant codes will be used during statistical analysis. Participant information will be stored in the EDC system at the Beijing Rehabilitation Hospital, Capital Medical University, China, for 5 years. The final dataset will be available to the principal investigator and independent statistician. The results of this study will be published in a peer-reviewed journal and presented at national and international conferences.

### Sample size

This is a randomised, double-blind, parallel, placebo-controlled clinical trial assessing the efficacy of iTBS among patients with PD. We used the primary outcome MDS-UPDRS III and considered that a plausible effect of the intervention would be a change in MDS-UPDRS III of − 4.89 points (− 3.25 points is for detecting minimal clinically important improvement difference on the Motor Examination part of MDS-UPDRS [62]). Values of mean (SD) for MDS-UPDRS III in control group will be 4.88 [63].

Based on a 1:1 ratio of patients in the iTBS + PT and Sham-iTBS + PT groups, the minimum sample size was calculated with formula shown in Eq. (1), providing 90% power with a level of 0.05.

1 $$n= \frac{{{(Z}_{a}+ {Z}_{\beta })}^{2} \times 2{\sigma }^{2}}{{\delta }^{2}}$$

In Eq. (1), *n* represents the sample size of each group, *σ* represents the values of mean (SD) for MDS-UPDRS III in the control group, *δ* represents the difference between the mean of the iTBS + PT group and the Sham-iTBS + PT group; *α* represents type 1 errors and *β* represents type 2 errors.

Application of Eq. (1) showed that 21 are required for the iTBS + PT group and the Sham-iTBS + PT group, respectively. Considering a 12% dropout rate, we plan to recruit 50 cases for the two groups.

### Statistical analysis

SPSS v22.0 software (SPSS Inc., Chicago, IL, USA) will be used for data processing and analysis. Measurement data are expressed as mean ± standard deviation. The intention-to-treat (ITT) analysis will be the primary analysis of this study, and per-protocol(PP) analysis will be a secondary analysis if there are patients that did not receive the intervention because of problems of the intervention itself: adverse effects, difficult administration or adherence, etc. The missing data will be processed using the last observation carried forward (LOCF).The sensitivity analyses will be used if there are unbalanced losses to follow up or if too many losses, etc. The significance level for each test will be set at *P* < 0.05.

MRI pre-processing will be carried out using the data processing assistant for Rs-fMRI, which runs with the statistical parametric mapping software (SPM82) [64]. After the EEG data is recorded, it is processed offline through MATLAB software scripts and the EEGLAB toolbox [64].

### Patient public involvement

We encourage patients and public collaborators been actively involved in the development of the protocol. The PPI contributors worked with the study team to design participant facing materials (study consent form) and contributed to the study design and management and develop the interview topic guides, interview participants, analyse data and disseminate findings (e.g. attending conferences, delivering lectures and seminars and co-authoring papers for publication).

## Discussion

PD is a common neurodegenerative disease with a prevalence of 1.7% in people over the age of 65 [65]. As the life expectancy of the world population increases, the prevalence and incidence of PD are expected to double by 2030 [66]. Currently, there is no complete cure for PD. In addition to drugs and DBS surgery, effective rehabilitation interventions can effectively improve the motor function, cognitive function and quality of life of patients with PD.

In recent years, rTMS has gradually become an important and effective method in the treatment of PD, which can be used to regulate cerebral cortex excitability and neuroplasticity in PWP [23, 24, 27]. TBS is a new model of transcranial magnetic stimulation (TMS). Intermittent TBS (iTBS) is believed to have more potent excitatory effects and can improve the induction of synaptic LTP in a much shorter time than traditional stimulation (5 min versus 30 min) [19, 22, 67]. There is increasing evidence that TBS is safe, has a shorter single treatment time and has a lower stimulation intensity, avoiding overheating the coil, resulting in more durable cortical excitatory effects and other advantages. A recent study identified that the use of “frequency oscillations external stimulation,” based on the brainwave frequency inherent to the brain area, significantly improved the function of the target [18, 20, 26, 68].

There are a variety of stimulation sites that can be selected for rTMS: the M1, SMA, DLPFC and cerebellum. One meta-analysis found that rTMS may contribute to improved motor function in patients with PD. In particular, HF-rTMS has a more significant effect than LF-rTMS; stimulation above M1 is more effective than stimulation at other parts; bilateral stimulation is more effective than unilateral stimulation; multiple courses and higher doses of pulses (18,000–20,000 pulses in total) are associated with better exercise outcomes [69].

The purpose of physical therapy is to help individuals maintain their maximum level of activity and independence. Physiotherapy as an effective strategy for improving gait, overall physical function and quality of life in PWP has been validated by systematic reviews and guidelines [70-73]. Balance-training exercises are known to improve postural control and equilibrium in PWP [44, 45] and are appropriate for challenging sensorimotor integration mechanisms [47, 48]. Some studies reported increments in walking velocity with treadmill training [74, 75], which is considered the standard intervention for gait rehabilitation [76].

To our knowledge, this is the first study to address motor function and quality of life for PD through PT, based on altering the neuroplasticity of motor-related brain regions through iTBS. The research uses iTBS stimulation as a tool to regulate the functional state of brain regions before PT, which is currently a well-documented and effective method for altering brain plasticity [77, 78]. We hypothesise that iTBS stimulation could alter brain plasticity, thereby promoting the efficacy of PT for PD. This study uses a randomised, double-blind design to validate the effectiveness and long-term effects of iTBS on quality of life and overall function in patients with PD in the context of PT and setting up rTMS treatment and sham control groups. The ultimate goal is to associate the behavioural improvement effects of iTBS and PT on mild to moderate PD patients with changes in the functional connection of the frontal lobe-striatum neural network and the biomarkers of neurobiological plasticity, using structural and functional magnetic resonance imaging, combined with BDNF levels at different time points before and after the intervention. These results can establish a normalised model for PD rehabilitation and may even be applied to other neurodegenerative diseases, while providing new hope for patients to live longer, be healthy, independent and improve their quality of life.

## Trial status

Patient recruitment is currently ongoing. This is the second version of this protocol (V2.0). After passing the ethics committee review, a copy of the revised protocol will be sent to the managers of the centre and be added to the web for accurate records. The first patient was enrolled on April 14, 2022, and it is expected to finish by the end of 2023. Due to the impact of COVID-19, our related studies have been severely affected, and the end time will be appropriately postponed.

## Supplementary Information

**Additional file 1.** Spirit checklist.

## Data Availability

The datasets are available from the corresponding author on reasonable request.

## Abbreviations

iTBS: Intermittent theta-burst stimulation
LEED: Levodopa equivalent dose
LTD: Long-term depression
LTP: Long-term potentiation
MDS-UPDRS: Movement Disorders–Unified Parkinson’s Disease Rating Scale
MRI: Magnetic resonance imaging
NMSS: Non-motor symptoms Scale
PDQ-39: Parkinson’s disease questionnaire
PT: Physiotherapy
PWP: Patients with Parkinson’s disease
rTMS: Repetitive transcranial magnetic stimulation
